# Bio-Inspired Multi-Functional Drug Transport Design Concept and Simulations †

**DOI:** 10.3390/bioengineering4020037

**Published:** 2017-04-25

**Authors:** Ramana M. Pidaparti, Charles Cartin, Guoguang Su

**Affiliations:** 1 College of Engineering, University of Georgia, Athens, GA 30602, USA; 2 Department of Mechanical and Nuclear Engineering, Virginia Commonwealth University, Richmond, VA 23284, USA; cartincp@vcu.edu; 3 Previously at Department of Mechanical and Nuclear Engineering, Virginia Commonwealth University, Richmond, VA 23284, USA; Guoguang_su@yahoo.com

**Keywords:** molecular motors, drug delivery, computer-aided-design, analysis, simulation

## Abstract

In this study, we developed a microdevice concept for drug/fluidic transport taking an inspiration from supramolecular motor found in biological cells. Specifically, idealized multi-functional design geometry (nozzle/diffuser/nozzle) was developed for (i) fluidic/particle transport; (ii) particle separation; and (iii) droplet generation. Several design simulations were conducted to demonstrate the working principles of the multi-functional device. The design simulations illustrate that the proposed design concept is feasible for multi-functionality. However, further experimentation and optimization studies are needed to fully evaluate the multifunctional device concept for multiple applications.

## 1. Introduction

Nature’s nanomachines include molecular pumps, motors, and sorters. They are the essential agents of movement and are integral parts of many living organisms. Nature’s molecular structures such as NPC (nuclear pore complex) are multifunctional, and are far more efficient than any man-made sensors/actuators. The NPC senses, actuates, and controls the transport of all cellular material between the cytoplasm and the nucleus, and this process occurs in all biological cells of many organisms including yeast, vertebrate, and others. In the presence of appropriate chemical stimuli, the NPC opens or closes, like a gate, and permits and modulates the flow of material into and out of the nucleus proteins by biochemical interactions, ion potential, and hydrodynamic transport [1,2,3,4,5,6,7,8,9,10]. The actual dynamics of molecular transport across the NPC are not completely known. Due to the NPC’s complex architecture, the structural changes as well as property changes during transport are not completely understood. Even though the complete structure–function of this biological motor is not understood completely, our objective is to take inspiration from this biological motor and investigate a design concept for multifunctional applications.

In recent years, several microdevices for fluidic transport have been developed. These include drug delivery systems [11,12,13], insulin injectors [14], fuel cells [15], space missions [16,17], and macromolecule and cell analysis [18]. Due to the NPC’s interesting and unique geometric architecture, various components play an important role in controlling the transport of material. Even though there are several unknowns in the NPC structure–function relationship, we believe that taking inspiration from its functions will lead to novel design solutions and assist in designing nano/micro-scale machines for mechanical and fluidic transport in engineering applications.

In this study, we developed a microdevice concept involving a nozzle/diffuser/nozzle configuration (grossly idealizing a biological motor geometry) for drug/fluidic transport. The specific advantage of developing a device concept inspired by an idealized NPC is that one geometrical configuration can be used for multiple applications (fluidic/particle transport; particle separation; and droplet generation). In addition, the design is also bidirectional similar to NPC and can achieve specific design efficiencies for different applications. The design configuration can also be optimized for reducing the backflow in comparison to nozzle/diffuser designs that exist in the literature.

Idealized multi-functional design geometry with actuating walls was developed for (i) fluidic/particle transport; (ii) particle separation; and (iii) droplet generation. Several design simulations were conducted to demonstrate the working principles of the multi-functional device. The present manuscript is a summary of the previous research investigations by the authors. In previous publication, the device concept (nozzle/diffuser/nozzle) was demonstrated through simulations for each aspect of its functionality, fluid pumping [19], drug delivery [20,21], or particle sorting [22] applications. Once we demonstrated the feasibility of each of the applications, we felt that the device concept could be adapted to multiple applications, and that is the focus of the present paper.

## 2. Multi-Functional Device Design

**Figure 1:**
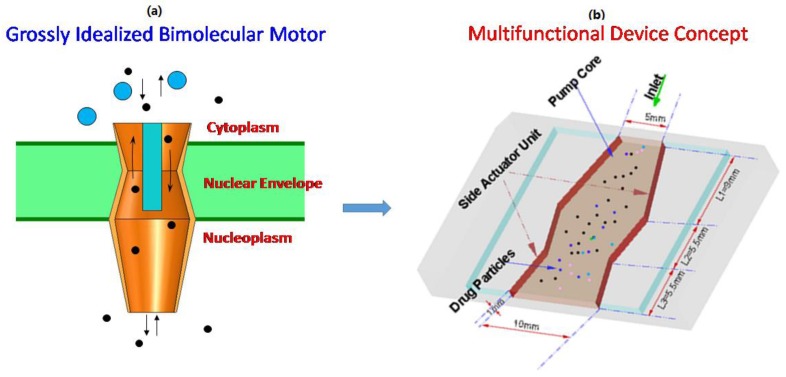
(**a**) Inspiration from grossly idealized biomolecular motor and (**b**) the developed multi-functional device design concept.

An idealized geometry representing of NPC containing the central plug, bottom basket, and top cytoplasm rings (nozzle/diffuser/nozzle elements) shown in Figure 1a, similar to the one described in the literature [4], was considered in this study. Kittisak et al. [19] demonstrated in computational studies that there are several advantages to using the three nozzle-diffuser microdevice as compared to the two nozzle-diffuser pump including minimization of backflow, more directed flow from inlet to outlet, steady flow velocities, and better laminar flow characteristics throughout the entire microdevice. The sidewall motion can be achieved either through piezoelectric or magnetic actuator or any combination. The device configuration was designed in such a way that it can pump the fluid into the air and encourage droplet breakup and aerosol formation as well as particle separation.

## 3. Design Analysis Methodology

The design methodology was developed to illustrate the multifunctional (fluidic/particle transport, particle separation, and droplet generation) functions of the microdevice. The details are briefly described below.

### 3.1. Design Computational Model

Design simulations based on finite element analysis was carried out for geometric models shown in Figure 1b. The microdevice design simulation for fluidic transport requires solving the Navier–Stokes equations related to conservation of mass, and momentum. The standard governing equations for the laminar flow are described as

(1) $$\frac{\partial_{\rho }}{\partial_{t}}+\nabla •\left(\rho \vec{V}\right)=0$$

(2) $$\rho \frac{D\vec{V}}{Dt}=-\nabla P+B+\mu \nabla^{2}\vec{V}$$

where $\vec{V}$ is the velocity vector, *P* is the pressure, *ρ* is the fluid density, *B* is the body force, and *μ* is its dynamic viscosity. The above equations were solved numerically on a fluid domain with moving walls to obtain the time-dependent flow field. A general-purpose computational fluid dynamics solver FLUENT software (ANSYS/FLUENT Inc., Canonsburg, PA, USA/Pittsburgh, PA, USA) [23] with the finite volume method was used to carry out the simulations, and the transient solution was implemented with implicit marching techniques. The moving mesh approach updates the actuating walls with a new discretized computational domain at every time step. The convergence criteria used is based on the number of iterations to achieve 10^−5^ for residuals of mass and momentum equations. To reduce the iteration error, the second-order accurate scheme was selected for spatial discretization. The SIMPLE algorithm was used for solving the pressure–velocity coupling, and this procedure is repeated at every time step until a converged solution for instantaneous flow field is obtained.

The movement of the microdevice actuation units to create a unit movement of periodic volume expanding and contracting is given by the following expression:

(3) $$s\left(x,t\right)=A\sin\frac{\pi x}{R}\sin2\pi \omega t$$

where s(x,t) is the displacement of the wall in vertical direction, and *ω* is the vibrating frequency of the microdevice chamber. Due to the unique sequence of operations involved in the actuation events of the microdevice, the expected operating mode of unit vibration actuation can change the flow direction as well as cause the net fluid to be “pumped” from one side to another side of the microdevice.

The particles movement in the flow field can be simulated by the equation of the particles motion [24], which is described by the following equations:

(4) $$\frac{d{\vec{V}}_{p}}{dt}=f\left(\vec{V}-{\vec{V}}_{p}\right)/\tau_{p}+g\frac{\rho_{p}-\rho }{\rho_{p}}+\vec{F}$$

(5) $$\frac{d{\vec{X}}_{p}}{dt}={\vec{V}}_{p}$$

In the above equations, $\vec{V_{p}}$ and $\vec{V}$ are the particle velocity vector and local fluid velocity vector, respectively. $\vec{X_{p}}$ is the particle trajectory obtained by integrating the kinematic and dynamic equations. $f\left(\vec{V}-\vec{V_{p}}\right)/\tau_{p}$ is the drag force on the particle, where τ*_p_* = *ρ_p_*·*d_p_*^2^/18µ is the characteristic time required for particles to respond to changes in the flow field. The drag factor *f*, which represents the ratio of the drag coefficient to Stokes drag, is based on the expression of Morsi and Alexander [24].

(6) f=CDRep24=Rep24(a1+a2Rep+a3Rep)

where *a_i_* coefficients are available for multiple particle Reynolds number ranges expected for the particles of interest.

For simulating the droplet generator aspect of the microdevice, the two-phase flow field (liquid phase in the microdevice and gas phase in the spray region) was solved using computational fluid dynamics with moving mesh technique by tracking the volume fraction of each of the fluids throughout the computational domain. For simulating the flow field in both the device and spray domains, the Navier–Stokes equations were solved under laminar, isothermal, and incompressible conditions. The continuum surface force (CSF) model proposed by Brackbill et al. [25] was adopted to address surface tension at the interface of the gas and liquid. Additional details of the computational methods, and the relative validation and calculation procedures used in the design simulations can be found in Su et al. [20].

### 3.2. Prototyping and Testing

**Figure 2:**
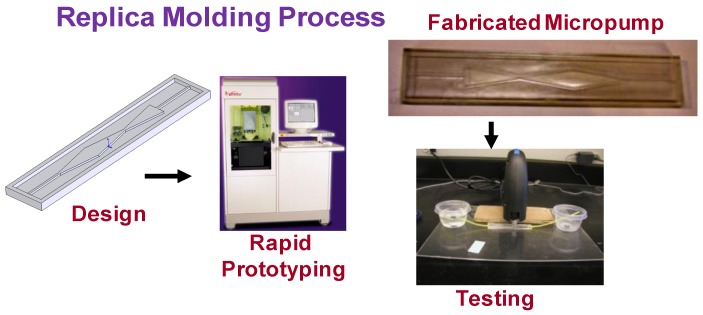
Prototyping the device for fluidic transport.

In order to illustrate the microdevice design concept for fluid pumping/transport, a prototype of the three-nozzle/diffuser microdevice was fabricated using polydimethylsiloxane (PDMS) material using standard rapid prototyping and CAD models as shown in Figure 2. Accura60 resin (3D Systems, Inc., Atlanta, USA) was used in preparing the microdevice mold and fabricated using SLA prototyping. The PDMS material was prepared and poured into the mold, and the chemical remover and cleaner was utilized to finish the mold using standard Denature Alcohol. The details of the microdevice fabrication can be found in Cartin et al. [26]. Due to difficulties involved in simulating and testing side actuation, testing was performed with top actuation. The specified actuation was achieved by using a reciprocal motor actuator that is accurately controlled with an externally supplied voltage. Water with a density of 998.2 kg/m^3^ and viscosity of 0.001003 kg/m·s was used as the working fluid in the microdevice. A pressure difference of 0 Pa was set for the boundary condition at the inlet and the outlet of the microdevice. No-slip boundary condition was applied at an interface between microdevice walls and the working fluid. The performance of the device with respect to the flow rate and optimum pumping frequency was evaluated by testing various fluids with different viscosities.

## 4. Results and Discussion

Design simulations were conducted to assess the drug transport, droplet generation, and particle separation features of the microdevice concept. The results obtained from these design simulations are briefly described below.

### 4.1. Drug Transport Design Simulations

**Figure 3:**
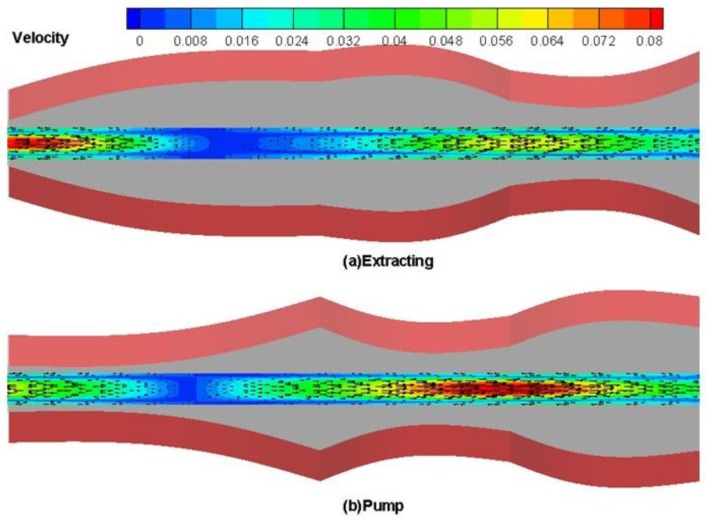
Fluid pumping through the device—instantaneous velocity vector contours at 50 Hz. (**a**) Extracting; (**b**) pumping.

**Figure 4:**
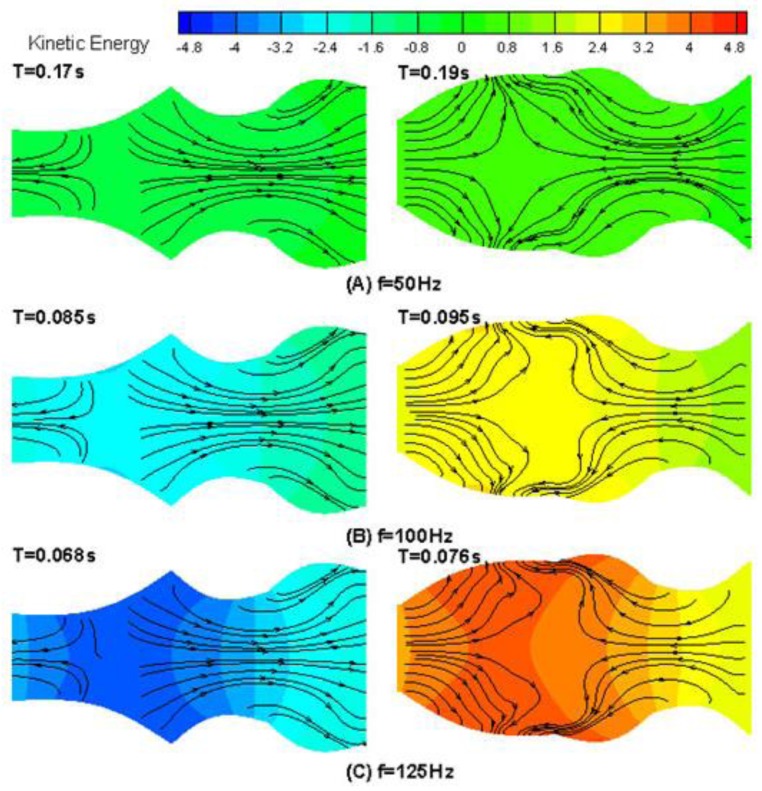
Fluid pumping through the device—instantaneous kinetic energy contours and streamlines in one period with different frequencies. (**A**) f = 50 Hz; (**B**) f = 100 Hz; (**C**) f = 125 Hz.

In order to illustrate the drug transport characteristics of the microdevice, the results of velocity vector and velocity magnitude contours in the middle cross section of the microdevice are presented in Figure 3. To further illustrate trends in the flow pattern, the instantaneous streamline and kinetic energy during one period of device chamber movement at different frequencies is shown in Figure 4. As the results presented in Figure 3 and Figure 4 clearly illustrate device actuation was achieved with two characteristic phases (extracting and pumping). As shown in Figure 3, the flow passageway near the inlet expands during extraction, and fluid flows into the microdevice from the inlet, while the passageway near the outlet expands during pumping mode and the fluids flow out. Similar behavior was observed for the streamlines and kinetic energy contours when the drug is transported through the device at different frequencies as shown in Figure 4.

**Figure 5:**
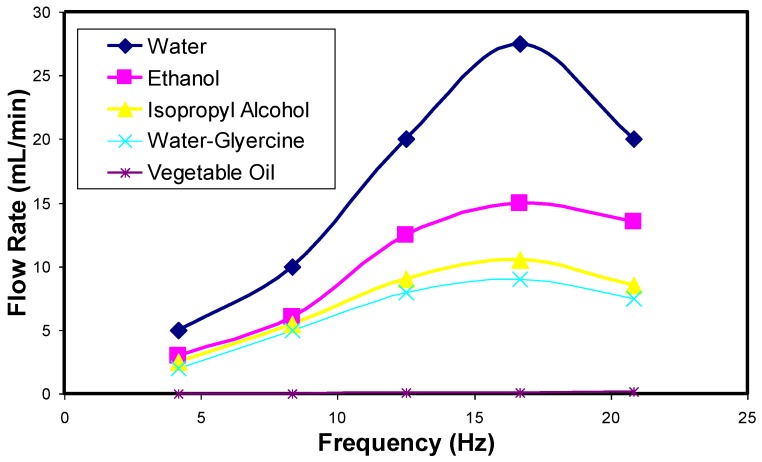
Flow rate through the designed microdevice for drug transport/pumping of different fluid media at various actuating frequencies.

The results of flow rate obtained with varying actuator strokes (frequencies) for different fluids are summarized in Figure 5. As shown in Figure 5, the flow rate for various fluids follows a similar pattern when flow rate is increased. The flow rate increases, reaches an optimum, and then decreases. Out of the five fluids tested, water with high density had a higher flow rate in comparison to other fluids. Unfortunately, we did not test the device with any body fluids or simulated buffers. However, the authors feel that the range of fluids tested will give an idea of the feasibility of pumping different fluids through our device. However, the pumping rates for particular specific fluids may be different. Overall, the results of design simulations and limited testing illustrate the utility of the microdevice for drug transport/pumping applications.

### 4.2. Droplet Generation Design Simulations

**Figure 6:**
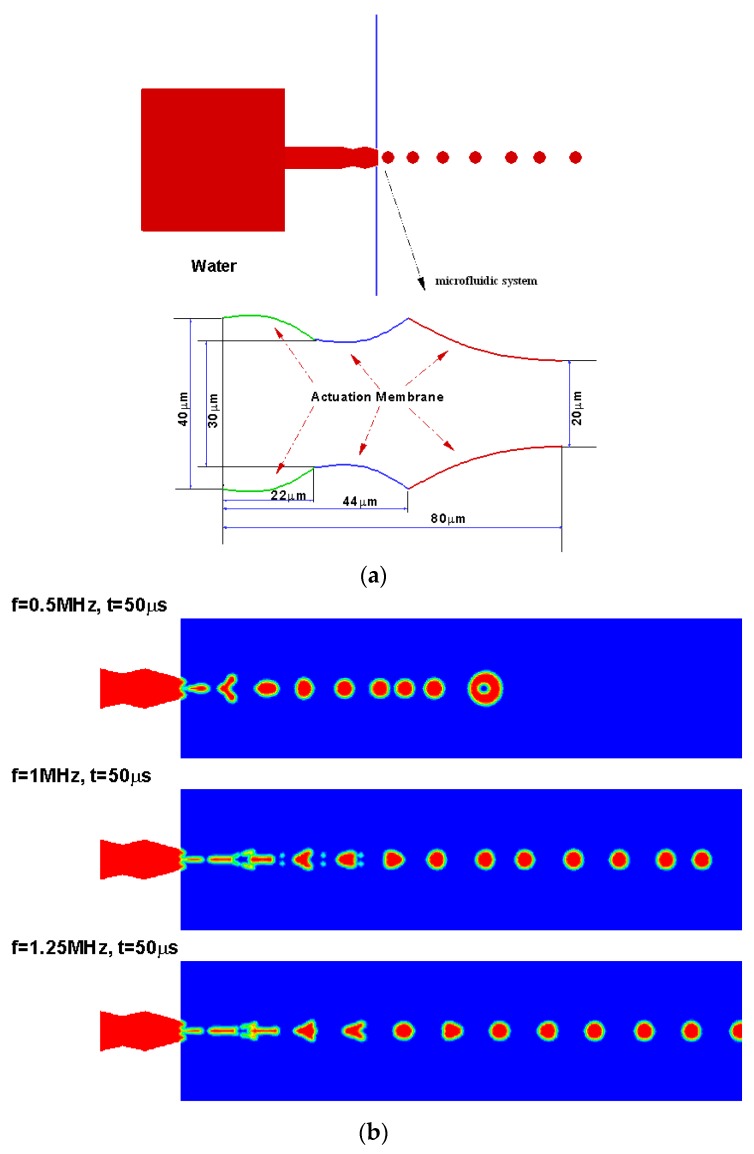
(**a**) Design concept for droplet generation using moving walls; (**b**) design simulations from a novel droplet generator. Reproduced from reference [20], with the permission of AIP Publishing.

A bioinspired structure with three-nozzle/diffuser elements with top and bottom walls moving was developed to produce monodisperse droplets on demand for aerosol drug delivery applications. The novel droplet generator microdevice is illustrated in Figure 6a. The device was designed to both pump fluid into the air and encourage droplet breakup and aerosol formation. The same microdevice used earlier in drug transport study was applied to eject the fluid to the surrounding air. It was expected that the oscillatory motion of the device walls would create additional instability in the fluid and produce breakup at lower Reynolds numbers than required for typical fluid-in-air jets.

Figure 6b shows the temporal evolution of liquid droplets ejected by the microdevice at various actuation frequencies for 50 ms. As shown in the illustration above, the microdevice droplets generator discharged monodisperse droplets continuously after an initial startup period. The first droplet, which was generated into a still environment, had a larger size and was slightly irregular. Following this lead droplet, subsequent droplets were highly uniform with a diameter equal to the exit size of the microdevice. It can also be observed that uniform droplets are ejecting from the outlet of the device. More results with design variations of the microdevice can be found in Su, Longest, and Pidaparti [20].

### 4.3. Particle Sorting Design Simulations

**Figure 7:**
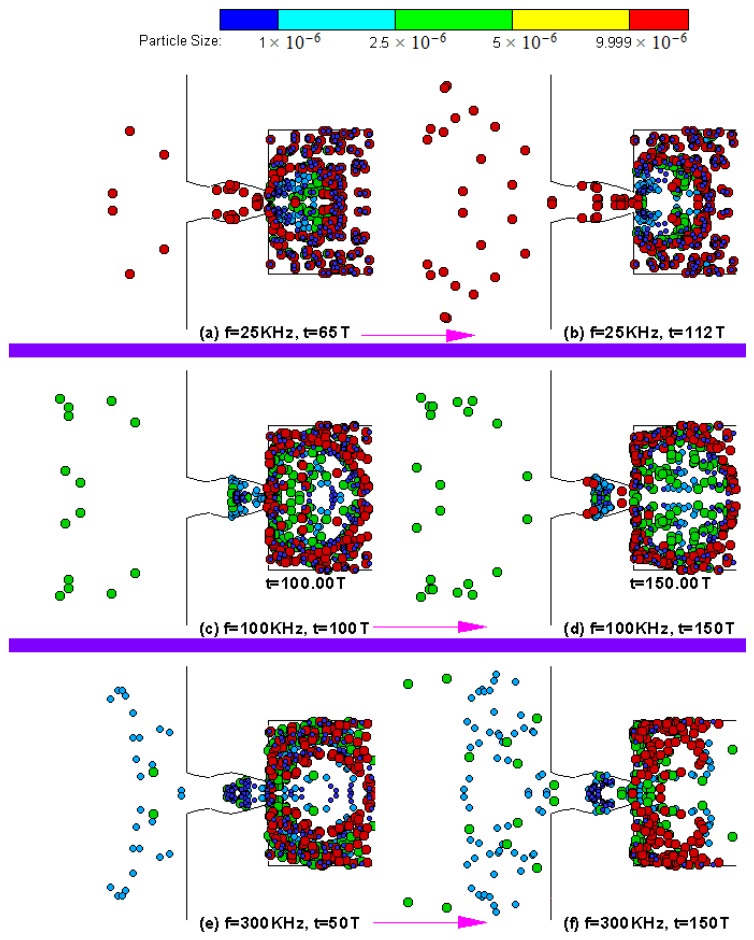
Design simulations of a particle separator device. (**a**) f = 25 KHz, t = 65 T; (**b**) f = 25 KHz, t = 112 T; (**c**) f = 100 KHz, t = 100 T; (**d**) f = 100 KHz, t = 150 T; (**e**) f = 300 KHz, t = 50 T; (**f**) f = 300 KHz, t = 150 T. Reproduced from reference [21], with the permission of ASME Publishing.

Based on an idealized geometry concept shown in Figure 1a, we also developed a device for particle sorting as shown in Figure 1b using time series alternate flow in the microfluidic device. The working principle of the proposed mechanism was demonstrated by a computational simulation using a device for separating the particles with a 1–10 μm diameter. Figure 7 shows the simulation results for particle separation. The instantaneous particle location at 50, 100, and 150 vibration cycles of the micropump actuation at 1000 kHz is shown in Figure 6. It can be observed that the larger size particles appeared in the left side of the micropump device at 50 T, while the small size particles (with 1 μm diameter) were clustered around the pump body. With continuous operation of the micropump, more particles with larger sizes entered into the receiver (left side), while the particles with a 1 μm diameter remained in the pump body at 100 vibration cycles and so on up to 150 vibration cycles. The results presented in Figure 6 indicate that the proposed mechanism can perform a separation of particles both spatially and temporally according to the particle size.

Figure 7 presents the *cumulative particle* percentages received at the outlet of the microdevice at various actuation frequencies. When the microdevice was actuated at 25 kHz, only particles with a 10 μm size accumulated at the outlet (Figure 7a,b), whereas, with increasing actuation frequency, particles of 10 μm and 5 μm accumulated at the outlet (Figure 7c,d), and so on. The temporal separation of the particles and the accumulation of different sizes at different frequencies is displayed in Figure 7. More results with design variations of the microdevice can be found in Su and Pidaparti [22]. The numerical results obtained indicate that the proposed design concept is feasible, and the optimized design (actuating frequency and the microdevice geometry) can be achieved for specific scientific applications. Although we demonstrated the feasibility of particle separation with a microdevice concept, the proposed device can be applied in other engineering applications as well.

In summary, a microdevice concept with a nozzle/diffuser/nozzle configuration with cascaded moving boundaries was developed for drug transport, droplet generation, and particle sorting. We conducted design simulations with Computational Fluid Dynamics (CFD) and limited prototyping/testing to demonstrate the multi-functional features of the device. Overall, the results presented indicate that the design concept is feasible and needs further work in prototyping as well as additional testing of the physical device for other engineering applications.

## 5. Conclusions

A multi-functional-device concept taking an inspiration from supramolecular motor found in biological cells was developed in this study. An idealized multi-functional design geometry involving nozzle/diffuser/nozzle configuration was developed specifically for (i) fluidic/particle transport; (ii) particle separation; and (iii) droplet generation applications. Several design simulations were conducted to demonstrate the working principles of the multi-functional device. The design simulations illustrate that the proposed design concept is feasible for multi-functionality. However, further experimentation and optimization studies are needed to fully evaluate the multifunctional device concept for multiple applications.
